# Additive Manufactured Sandwich Composite/ABS Parts for Unmanned Aerial Vehicle Applications

**DOI:** 10.3390/polym10111262

**Published:** 2018-11-13

**Authors:** Athanasios Galatas, Hany Hassanin, Yahya Zweiri, Lakmal Seneviratne

**Affiliations:** 1 School of Engineering and the Environment, Kingston University, London SW15 3DW, UK; thanasisglt@hotmail.com (A.G.); Y.Zweiri@kingston.ac.uk (Y.Z.); 2 School of Engineering, University of Liverpool, London EC2A 1AG, UK; 3 Department of Aerospace Engineering, Khalifa University Center for Autonomous Robotic Systems, Khalifa University of Science and Technology, P.O. Box 127788 Abu Dhabi, UAE; 4 Khalifa University Center for Autonomous Robotic Systems, Khalifa University of Science and Technology, P.O. Box 127788 Abu Dhabi, UAE; lakmal.seneviratne@ku.ac.ae

**Keywords:** FDM, composite, sandwich structure, CFRP, neural network, UAV

## Abstract

Fused deposition modelling (FDM) is one of most popular 3D printing techniques of thermoplastic polymers. Nonetheless, the poor mechanical strength of FDM parts restricts the use of this technology in functional parts of many applications such as unmanned aerial vehicles (UAVs) where lightweight, high strength, and stiffness are required. In the present paper, the fabrication process of low-density acrylonitrile butadiene styrenecarbon (ABS) with carbon fibre reinforced polymer (CFRP) sandwich layers for UAV structure is proposed to improve the poor mechanical strength and elastic modulus of printed ABS. The composite sandwich structures retains FDM advantages for rapid making of complex geometries, while only requires simple post-processing steps to improve the mechanical properties. Artificial neural network (ANN) was used to investigate the influence of the core density and number of CFRP layers on the mechanical properties. The results showed an improvement of specific strength and elastic modulus with increasing the number of CFRP. The specific strength of the samples improved from 20 to 145 KN·m/kg while the Young’s modulus increased from 0.63 to 10.1 GPa when laminating the samples with CFRP layers. On the other hand, the core density had no significant effect on both specific strength and elastic modulus. A case study was undertaken by applying the CFRP/ABS/CFRP sandwich structure using the proposed method to manufacture improved dual-tilting clamps of a quadcopter UAV.

## 1. Introduction

Unmanned aerial vehicles (UAVs), popularly known as drones, have significantly improved since they were first introduced in the twentieth century. They become a preferable technology in many applications such as military, agricultural, search and rescue, telecommunications, topography, mapping, and surveillance [1,2]. The growing advancement of UAVs depends greatly on innovation in many technologies such as control, computer technologies interoperability, communications integration, and advanced manufacturing. Innovative manufacturing techniques and materials have been developed to improve the performance of UAVs by producing high endurance and lightweight structures [3]. This can be achieved by the implementation of materials with high specific strength, high impact properties, and stiffness.

Carbon fibre-reinforced polymer (CFRP) composites are strong and light materials that include reinforcement from carbon fibres and a polymer matrix. The matrix is often a thermoset material such as epoxy resin, but thermoplastic materials, such as nylon, can also be used to hold and protect the fibres, while carbon fibres will distribute the loading stress to the structure [4,5]. CFRP composites are lightweight and strong materials, which hold a wide range of extraordinary properties including excellent fatigue, high specific strength, and corrosion resistance. Such properties allow the use of CFRP composites in many applications, such as automotive, aerospace, structural, containers, sports products, and robot arms. One remarkable use of CFRP in commercial aeroplanes is in Airbus A350 whereby 53% of the materials used in the airframe are made of CFRP composite [6]. Light sport equipment such as tennis rackets and carbon fibre bikes provide high performance solution while athletes continue to drive for an advantage in tools and equipment [7,8]. In Addition, Formula (1) cars are commonly using CFRP not only because of its lightweight and strength but also because the advantage of its look [9]. As a result, CFRP composites take a key role in the design and manufacture of UAVs [10,11,12,13,14]. Sandwich-structured composite is a unique class of materials that is manufactured by adding two thin skin layers to a thick and lightweight core. They offer a superior specific strength and stiffness when compared to monolithic composites. The core material is commonly cheaper and has lower strength and density when compared to the skin layers [15,16,17]. Typical core materials include foam and balsa wood. Foam can be fabricated using polymer or metal while it is further divided into open and closed pores structures. Honeycomb structures made of different materials including aluminium, aramid paper, and paper are also widely used as core material [18,19]. On the other hand, the skin layers reinforce and provide the sandwich structure with stiffness and strength. They can be made of steel, aluminium, or fibre composites [20,21,22].

Additive manufacturing (AM) has advanced very rapidly over the past years to allow the manufacturing of complex shaped parts from metals, ceramics, polymers, and composites making it one of the most promising technologies in the near future [23,24,25]. The number of applications of AM has drastically risen, from aerospace, automotive, and biomedical to hobbyist and enterprises to fabricate a wide range of products in the forms of prototypes and final parts [26,27,28,29]. However, one main limitation of this technology is the limited strength of printed parts. While metal based AM processes such as Direct Laser Fabrication (DLF), Selective Laser Melting (SLM), also known as laser powder bed fusion, and Electron Beam Melting (EBM) are becoming favourable and robust to fabricate parts with satisfactory strength, their initial and running costs are very high when compared with other AM processes. Fused deposition modelling (FDM) is one of the cheapest AM techniques that are based on material extrusion [30]. The availability and the low cost of FDM made it a widespread printing technology that many hobbyists have taken up and used for many purposes [31,32,33]. Thermoplastic materials are commonly used in FDM because it is easy to shape them into filaments with specific sizes. In addition, their melting temperatures are relatively low and their viscosities are suitable during extrusion and deposition. The most common materials used in FDM and their properties are presented in Table 1. As shown in the table, the materials are generally limited to options such as acrylonitrile butadiene styrenecarbon (ABS), Nylon, Poly Lactic Acid (PLA), and Polycarbonate, with strength between 26–56 MPa and elastic modulus between 1.2–3.6 GPa [32,34,35,36,37,38,39,40]. The low strength of its prints restricts its applications to produce functional parts.

Several approaches were developed to increase the strength of 3D printed plastic parts. Filling the voids of the 3D printed samples with high strength resin were found to effectively increase the mechanical strength and stuffiness by 45% and 25%, respectively [41]. On the other hand, ultrasonic strengthening technique was found to improve the tensile strength by about 22% [42], while localized infra-red laser heating was used to enhance the inter-layer interface temperature and hence to improve the bonding strength by about 50% [43]. As shown, efforts to increase the strength of 3D printed parts to be used in many load-bearing applications such as robotics and other domains has attracted many researchers. However, to the best of the authors’ knowledge to date the use of sandwich-structured composite to improve the mechanical properties of 3D printed plastic parts is lacking, which is the aim of this paper. In this paper, we introduce a novel method to improve the strength of 3D printed polymeric parts for the use in high performance applications. The research investigates the use of sandwich-structured composites with core material fabricated using fused deposition modelling laminate it with a thin CFRP skin. The experiments performed in this paper aimed to identify whether the use of CFRP/ABS/CFRP sandwich composites can have improved mechanical properties when compared to the monolithic 3D printed parts and what is the optimum number of CFRP layers to maximize the specific strength and the Young’s modulus.

## 2. Materials and Methods

### 2.1. Design of Experiment

Recently, different techniques were introduced to investigate the relationship between inputs parameters and responses, such as regression analysis and design of experiment. Regression analysis is a set of statistical methods to estimate the relationships between variables. However, the precision of regression technique is inadequate for predicting responses [22]. Design of experiments (DoE) is a statistical technique used to find the relation between input parameters and output results of a specific method aiming to optimise the results. The use of DOE has been shown to be a powerful tool to study the influence of process parameters in AM processes [44,45,46]. The response surface technique is a statistical DoE method to design and generate an approximate model and relation between the input and output process parameters [47]. In this study, it was aimed to improve the specific strength and the elastic modulus of the ABS printed samples by investigating the effect of the number of composite layers and the density of the ABS core as two process parameters. Table 2 shows the levels and range of the investigated process parameters.

### 2.2. Artificial Neural Networks

Artificial neural networks (ANN) have received a vast amount interest among researchers in the past two decades and they are considered as one of the best ever computational techniques [48]. The interest and the popularity in this tool for futuristic applications are because of their ability to mimic the brain’s functionality in learning and hence, they can take decisions for problems even those with incomplete data. In addition, it is an excellent statistical technique for numeric and nonnumeric situations [49]. ANN has helped in solving many complex problems in many areas such as energy, manufacturing, pharmaceutical, and engineering [50,51,52,53]. There are many forms of ANN; however, the most typical ANN layout developed by Rumehart et al. [53] is the back propagating network trained by error backpropagation. The input parameters provide information from the external source to a hidden layer through weighted connections. Next, the output layer generates results. The ability of ANN to organize data is influenced by the hidden layers, which are coupled with the neighbouring layers through synapses. The backpropagation algorithm for artificial neural networks searches for the minimum of a least square cost function using gradient descent. If the training model pairs: (I1, t1), (I2, t2), (I3, t3),…, (I*n*, t*n*) where I*i*, 1 ≤ *i* ≤ *n*, represent the *i*th input in the sample, and *ts*, 1 ≤ *i* ≤ *n*, is the target. The least square cost function in the networks weight space is:

(1) $$E=\frac{1}{nZx}\overset{n}{\underset{i=1}{\sum }}{\left[t_{i-}O_{i}^{X}\right]}^{\tau }\left[t_{i-}O_{i}^{X}\right],\;$$

where $O_{i}^{X}$ is the output vector of an X-layered network with Ii as input, and *Z_X_* is the number of output neurons.

#### 2.2.1. Backpropagation Algorithm

Assuming that *W* is a vector made by the weights of the network and ∇*E*(*W*(*h*)) is the derivative of *E* at *W* = *W*(*h*), with *k* = 1, 2, 3,…, *N*, are the iteration of the weights. The back-propagation algorithm with a momentum can be described as:

(2) ΔW(h)=α(−∇E(W(h)))+βΔW(h−1), 

where α is the learning rate, and β is and momentum factor, while Δ*W*(*h*) = *W*(*h* + 1) − *W*(*h*).

#### 2.2.2. Three-Term Backpropagation Algorithm

**Figure 1:**
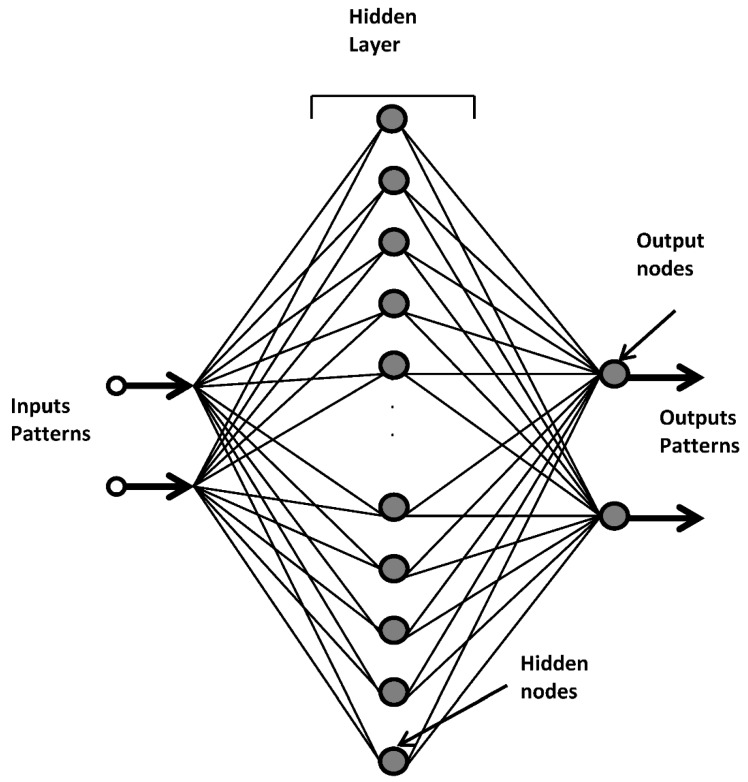
A schematic of forward artificial network. Input patterns receive data from input parameters and transfer it to the hidden layer with weighted connections. Output patterns provide the representative output results.

The BP algorithm described in Equation (2) was modified by the addition of an extra term so that the BP learning speed increases, less training data and to achieve a high performance [54,55]. The extra term is proportional to the difference between the output and the target at each iteration. The three-term BP algorithm in [49] is given as:

(3) ΔW(h)=α(−∇E(W(h)))+βΔW(h−1)+γe(W(h)), 

where γ is the proportional factor. It is noted that the BP algorithm described in Equation (3) has defined by three terms, first, a proportional to the ∇*E*(*W*(*h*)), second, a proportional to the preceding value of the incremental weights difference and a third term is proportional to *e*(*W*(*h*)). In this study, two third of sandwiched material properties data are chosen representing the output, and utilised for training. Each input is related with a particular output value. A neural network with two inputs, one hidden layer having 20 nodes, and 2 outputs are implemented. A typical neural network schematic diagram is shown in Figure 1. In Figure 1, the inputs patterns contain the sandwiched material density level and number of layers data sets while the outputs patterns contain the measured set of elastic modulus and specific strength. The hidden layer, which connects the input to the output are comprised of weight connections, hidden nodes and biases. Each hidden node is triggered by the activation function, while each output node is triggered by the linear activation function.

### 2.3. Experimental

ABS thermoplastic filament type UP Fila 500 g drum was supplied by (UP 3D printers, Kingston, UK), while the carbon fibre reinforcement fabric Pyrofil TR30S 3K with 0.m thickness was supplied from (Easy composites, Staffordshire, UK). The mechanical properties of the carbon fibre fabric are presented in the Table 3. El2 laminating epoxy resin and AT30 hardener were also supplied from (Easy composites, Staffordshire, UK).

**Figure 2:**
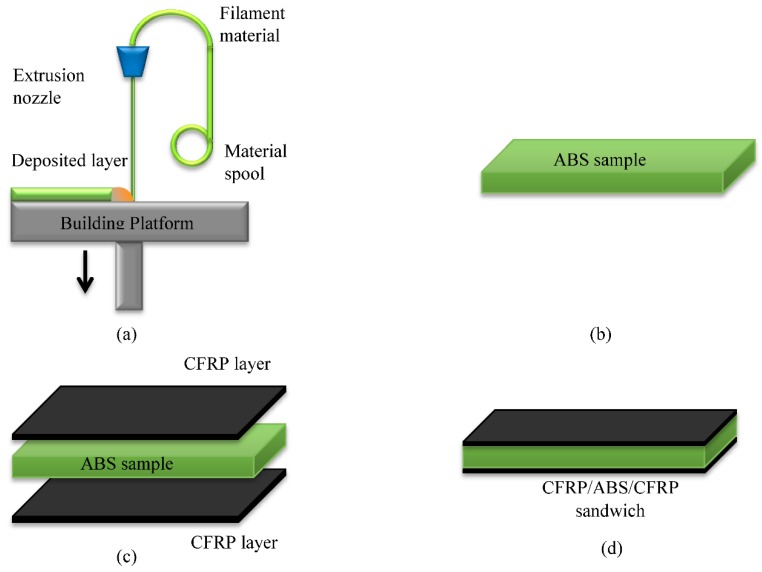
A schematic of the fabrication of CFRP/ABS/CFRP sandwich composite structure (**a**) FDM process; (**b**) ABS sample; (**c**) laminate the ABS with CFRP layers; (**d**) CFRP/ABS/CFRP sandwich.

The manufacturing procedure of CFRP/ABS/CFRP sandwich samples is shown in Figure 2.

**Figure 3:**
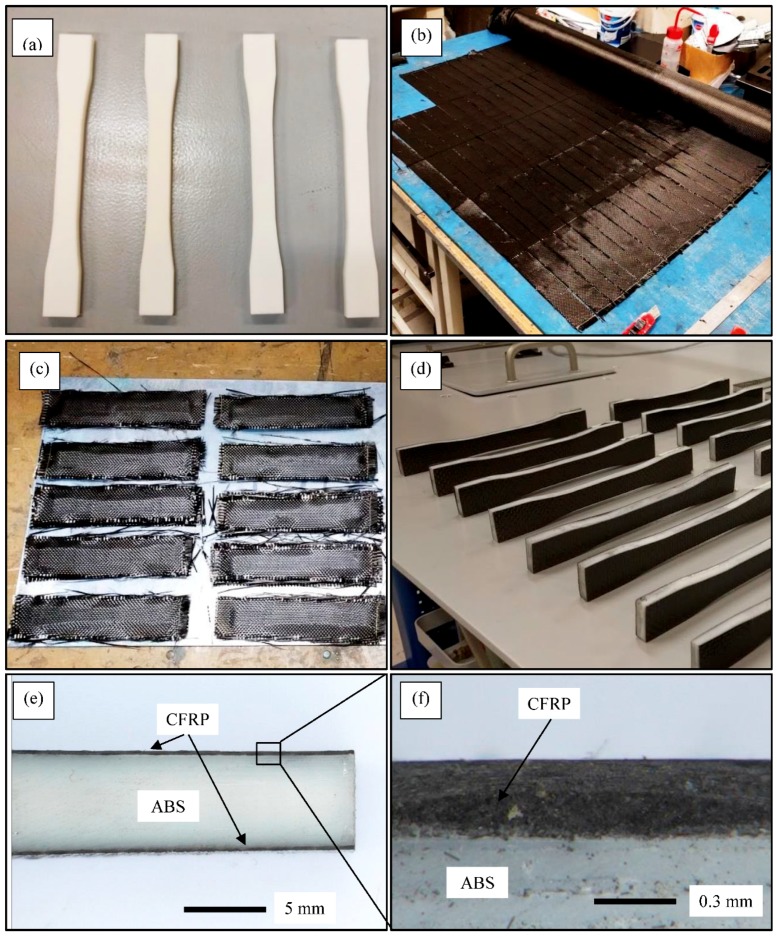
(**a**) ABS 3D printed test samples; (**b**) cut the woven CFRP according to the dimension of each samples; (**c**) glue CFRP layers on ABS surface and left to cure; (**d**) ABS/CFRP/ABS sandwich test samples ready for tensile properties measurements; (**e**,**f**) Optical microscopy images showing the ABS/CFRP interface at different magnification.

The FDM of ABS 3D samples started with creation of a CAD file representing the physical part, then conversion of the file using slicer software, uploading the file to the printer, and finally printing the object. All 3D printed parts were fabricated using UP BOX printers with a nozzle size of 0.4 mm, a layer thickness of 0.1, and the same printing orientation for each set in order to achieve identical printings. The only difference between these samples was the infill density. Three different densities were chosen for printing of the samples and they were 65% (designated as high), 56% (designated as medium) and 47% (designated as low). The ABS printed samples were first sanded to promote the adhesion with the composite layers. Afterwards, the woven CF layers were cut in dimensions similar to the printed samples. Next, the resin was mixed with the hardener with a ratio of 10 to 0.3 and applied to the woven CF of each sample. The layers were then bonded on both sides of each sample to form a sandwich structure. Finally, a load was placed on the surface of each sample to ensure a good bonding and removing any cavities and bubbles between the composite layers and the 3D printed samples then they were left to cure at room temperature for 24 h, see Figure 3a–d. The interface between the CFRP layers and the ABS printed samples were inspected under optical microscopy and the results are shown see Figure 3e,f. The figures show a good bonding between the CFRP layers and the core material. No gaps or cracks were observed which indicate that the process produces well bonded sandwich samples.

Densities of the 3D printed ABS samples were measured using the mass to volume ratio. Electronic scale with 0.001 g accuracy was used to measure the masses of the resin, hardener and the ABS samples. The tensile testing was carried out using Zwick/Roell Z050 machine (Kingston, UK). Tensile test samples were printed and tested according to ASTM D 638 standard. An extensometer epsilon model 3542 was used, it was connected to the tensile machine to calculate the deformation of the samples. The ultimate tensile strength, ductility and elastic modulus were measured for all samples. In addition, specific strength, also defined as the strength-to-weight ratio, was determined by dividing the material’s strength by its density.

### 2.4. Results and Discussion

#### 2.4.1. Mechanical Properties of CFRP/ABS/CFRP Sandwich Structures

**Figure 4:**
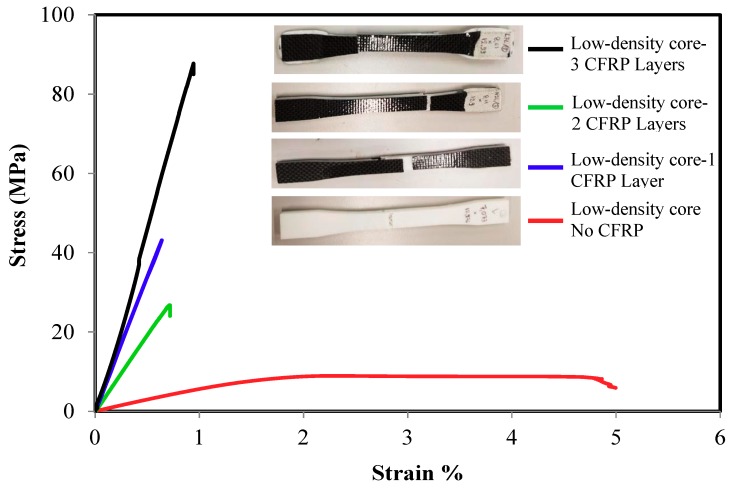
Typical tensile stress-strain response of low-density core CFRP/ABS/CFRP composite samples.

**Figure 5:**
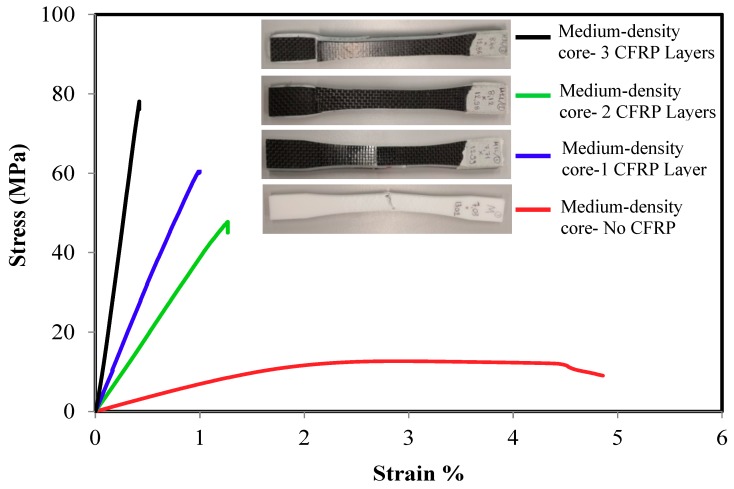
Typical tensile stress-strain response of medium-density core CFRP/ABS/CFRP composite samples.

**Figure 6:**
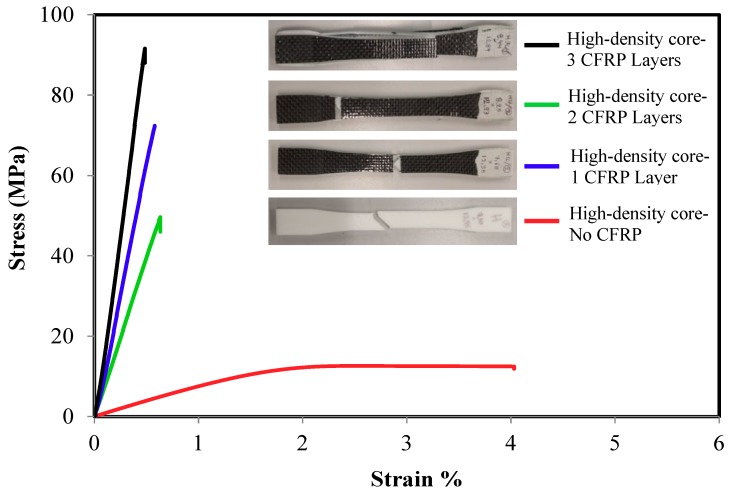
Typical tensile stress-strain response of high-density core CFRP/ABS/CFRP composite samples.

The tensile strength of the sandwich composite structure for different core density and number of layers are presented in Figure 4, Figure 5 and Figure 6. In general, all the three density samples showed a rapid increase in both the ultimate strength and the elastic modulus with the lamination of CFRP layers. Samples with monolithic ABS showed a ductile response while all the sandwiches samples exhibited brittle failure. Figure 4 compares the stress strain curves of one group of low density core ABS samples with 0, 1, 2, and 3 layers. The monolithic low-density core samples show the lowest ultimate strength, the lowest elastic modulus and the highest ductility. The figure shows a rapid increase in the materials’ strength with the addition sandwich CFRP layers. The addition of one layer CFRP on the top and the bottom of the ABS samples increased the average ultimate strength from 9.23 ± 0.34 MPa to 38.2 ± 3.9 MPa while the elastic modulus increased from 0.63 ± 0.1 GPa to 4.03 ± 0.7 GPa. On the other hand, samples with two CFRP layers had an increased ultimate strength of 43 ± 2.3 MPa while the elastic modulus increased to 7.3 ± 0.7 GPa. Furthermore, samples laminated with three CFRP layers increased the average ultimate strength to 93.3 ± 1.5 MPa while the elastic modulus increased to 10.13 ± 0.1 GPa. Similar results were achieved with medium and high-density core samples as shown in Figure 5 and Figure 6. For the medium-density core samples laminated with 3 CFRP layers, the highest strength and elastic modulus were 83.3 ± 1.5 MPa and 11.2 ± 1.7 GPa, respectively. Finally, the high-density core samples laminated with 3 CFRP layers exhibited a strength and elastic modulus of 93.2 ± 2.6 MPa and 10.2 ± 0.52 GPa, respectively. In summary, the ultimate strength improved about nine times and the Young’s modulus increased about 16 times as compared to the as printed samples.

**Figure 7:**
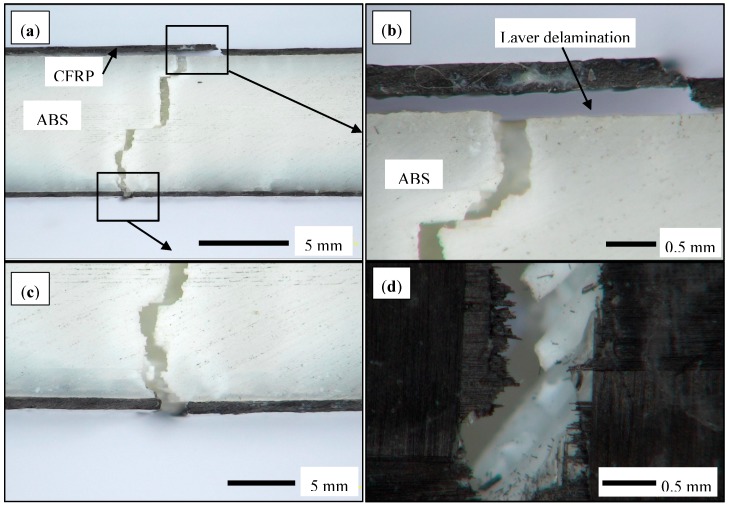
Optical microscopy images of the fractured tensile CFRP/ABS/CFRP sandwich samples: (**a**) side view of the broken sample; (**b**) top CFRP layer; (**c**) bottom CFRP layer; and (**d**) top view of the broken sample.

It was noticed that the failure of the sandwich CFRP/ABS/CFRP started at the CFR layers followed by the failure in the core material. This because the ductility of the CFRP is less than the ABS material. Figure 7 shows the tensile fractured samples. As shown, the failure of the core occurs close to the failure of the CFRP layer. The failure mode of the fiber layers were mixed between fiber pullout and CFRP layer delamination [56]. This type of failures occurred to the samples either individually or in combination; for example, part of the failure showed layer delamination, see Figure 7a,b, while the fiber layout for the CFRP layer and the ABS printed filaments was evidence in other regions as shown in Figure 7c,d. This indicates that although the samples exhibited a significant increase in the tensile strength and Young’s modulus, the layer delamination which occurs at a stress close to the failure. Using a vacuum bag and an autoclave may help to diminish CFRP layer delamination at high stresses. Methods such as filling with resin [41], ultrasonic strengthening [42], or using localized IR laser [43] improved the strength in a range of 22–50%. On the other hand, the CFRP/ABS sandwich technique that we proposed in this paper was able to achieve an increase in the tensile strength of about 9 times and an improvement of the Young’s modulus of about 16 times.

#### 2.4.2. Generation of the Experimental Matrix

The generated matrix of the nine process parameters and the corresponding results of the ABS/CFRP/ABS sandwich are shown in Table 4. The data listed in the table confirm the results obtained from the stress strain diagrams. In general, the results indicate a rapid increase in the specific strength and elastic modulus with the addition of CFRP layers. The specific strength and the elastic modulus of the monolithic samples were found in range of 19–23 kN·m/kg and 0.63–0.84 GPa, respectively. These properties were improved with the addition of CFRP layers. In particular, sample 7 prepared with low-density core and 3 laminated layers has the highest specific strength of 145 kN·m/kg, while sample 1 prepared using medium core and 3 laminated layers showed the best elastic modulus of 11.2 GPa.

#### 2.4.3. Response Surface and Artificial Neural Network Analysis

The response surface for specific strength and elastic modulus can be expressed as a second order polynomial equation as the followings. Using statistical software (Minitab), the constant values of the regression equation were calculated using collected data listed in Table 4. The response surface regression analysis resulted in the following predictive equation:

(4) $$\;{\mathrm{Responce}\;}_{\left(\mathrm{Infill}\;\mathrm{density}\right)}=b_{0}+b_{1}\left(\mathrm{No}\;\mathrm{of}\;\mathrm{layers}\right)+b_{3}{\left(\mathrm{No}\;\mathrm{of}\;\mathrm{layers}\right)}^{2}\;$$

where $b_{0},\;b_{1},\;b_{3}$ are the response surface coefficients where their values are presented in Table 5.

**Figure 8:**
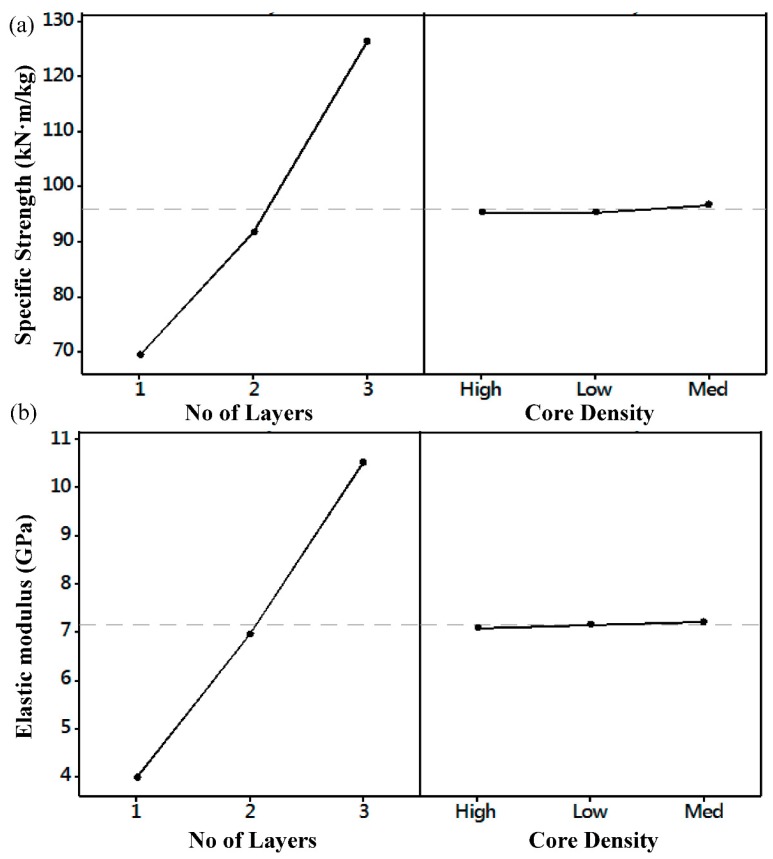
Effect of core density and number of CFRP layers on: (**a**) Specific strength; and (**b**) elastic modulus.

The model fit, *R*^2^ value, showed that the models described the relationship between process parameters and both the elastic modulus and the specific strength were 99.26% and 87.31%, respectively. Effect of the process parameters was further investigated and the material response was found as a function of core density and number of CFRP layers as shown in Figure 8. Analysis of specific strength and elastic modulus shows a clear dependence of these two responses on the number of layers. Specific strength and elastic modulus improve as number of layer decreases. On the other hand, these two responses are clearly independent with the core density. According to the surface response model, the specific strength and elastic modulus do not depend on the core density but they are greatly affected by the number of layers. 

**Figure 9:**
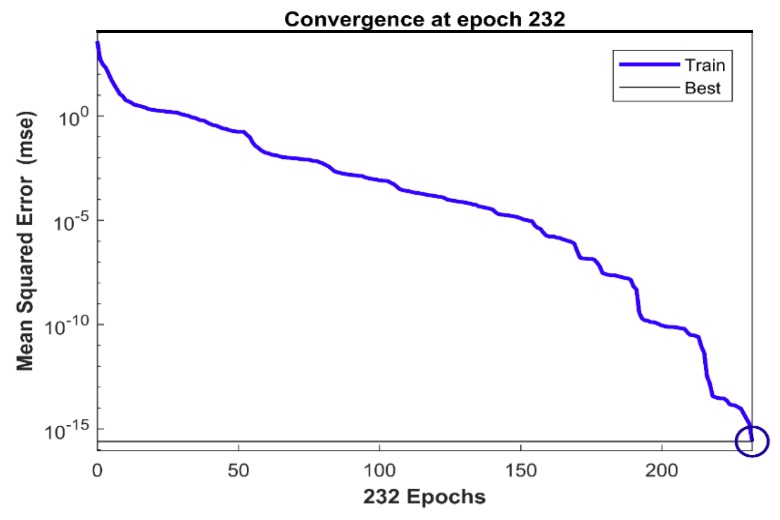
The conversions of the artificial neural network (ANN) training.

As described, the training of the neural network was carried out on the two third of the data listed in Table 4. In particular, results from the low and high-density core samples were only used in the training process of the model while results from the medium density core samples were excluded and used for validation. The high, medium and low-density core samples were designated as [+1, 0, −1] while the number of layers were set as [1, 2, 3]. The cost function was set as the mean square error and minimised by updating the results through the obtaining the unknown weight coefficients. The best solution was found after only 232 iterations, the neural network was converged to almost zero error, less than $10^{-15}$, as shown in Figure 9.

**Figure 10:**
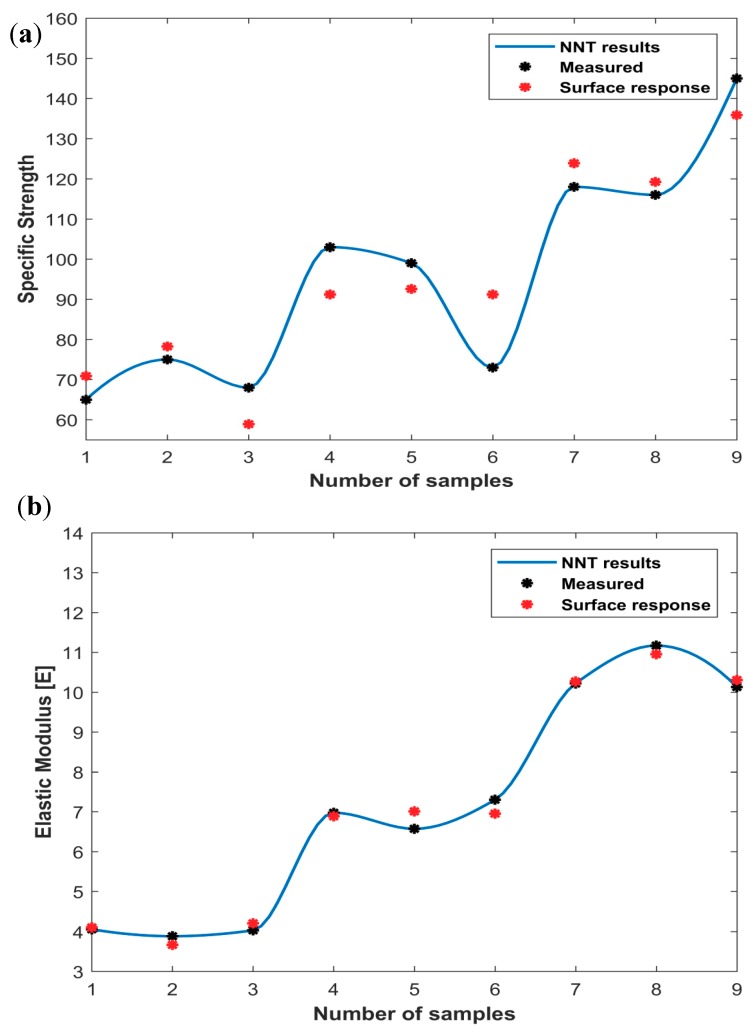
Measured results compared to the surface response and ANN models: (**a**) specific strength; and (**b**) elastic modulus.

The diagrams of the predicted surface response and ANN models versus the measured data are shown in Figure 10 to verify the accuracy of the two calculated models. As shown in the figure, there is an agreement in the trained ANN model with the experimental results. On the other hand, the ANN results are more accurate than the surface response for both the specific strength and the elastic modulus. In addition, the optimal ANN model provides errors of 0.63% and 0.48% for the specific strength and the elastic modulus, respectively. On the other hand, the surface response model gives errors of 10.26% and 3.23% for the specific strength and the elastic modulus, respectively. The ANN model has a better accuracy than the surface response model because ANN has the ability to map efficiently between the input and output by a powerful approximation of nonlinear relations and thus has better performance.

#### 2.4.4. Application on Dual-Tilting Clamp of Quadcopter UAV

**Figure 11:**
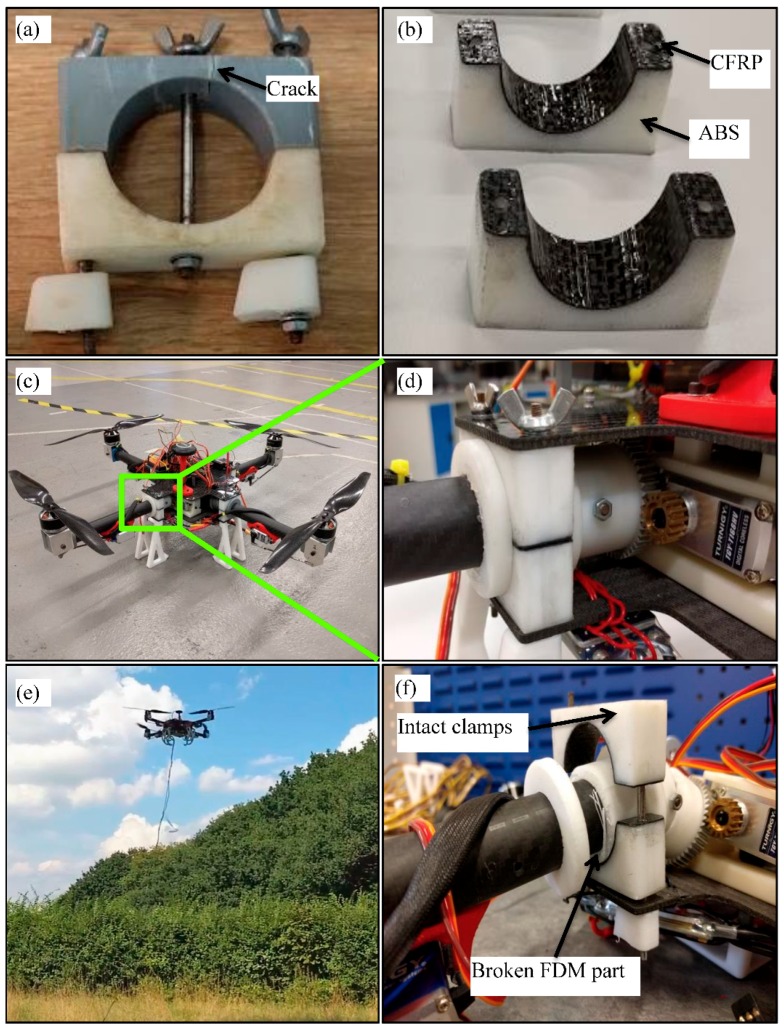
(**a**) Original monolithic clamp; (**b**) CFRP/ABS composite clamps; (**c**) Assembled quadcopter; (**d**) magnified photo of the assembled clamp; (**e**) quadcopter during flights test; (**f**) CFRP/ABS composite clamp after a crash test.

The developed CFRP/ABS composite structure with low-density core and three CFRP layers was used to reinforce the clamps of dual-tilting quadcopter UAV developed at Kingston University, London. Monolithic 3D printed ABS clamps were previously used and tend to break even after limited use, see Figure 11a. In addition, the monolithic clamps were getting loose due to wear, which had a negative effect on the stability of the platform. To solve this problem, new clamps were 3D printed in appropriate dimensions and reinforced with CFRP layers. The developed CFRP/ABS composite clamps using the proposed fabrication process are shown in Figure 11b. In addition, an overview of the whole platform with mounted CFRP clamps is shown in Figure 11c,d. The new CFRP clamps proved to be durable as were tested during several flights and rough landings, see Figure 11e. In addition, the quadcopter was tested in high impact crash from a height of 10 m on a hard floor. In this test, most of the monolithic 3D printed parts broke except the clamps reinforced by CFRP, which were intact proving their improved properties, see Figure 11f.

## 3. Conclusions

This paper proposed a novel sandwich structure with improved strength using additively manufactured ABS laminated with CFRP layers. It was aimed to improve the specific strength and stiffness of the resultant sandwich structure to meet the requirements of UAV applications. The paper also focused on the properties prediction and the relationship between process parameters and the properties of ABS/CFRP/ABS sandwich structure using both surface response and neural network analysis. A series of experiments were carried out by applying tensile measurements for 3D printed samples with different infill densities and number of CFRP layers. It was found that the ultimate strength and stiffness were significantly improved with applying CFRP sandwich layers. Under tensile loading, the ABS/CFRP/ABS sandwich structure exhibit behaviour brittle, while it was ductile for monolithic ABS samples. The ultimate strength improved about nine times and the Young’s modulus increased about 16 times as compared to the as printed samples. A surface response model was established to predict the material properties from the process parameters. The adequacy of the surface response model was verified. In addition, a nonlinear predictor based on artificial neural networks is developed to predict the elastic modulus and specific strength of composite material. The predicated results of the developed artificial neural networks have an excellent accuracy when compared to experimental data. Due to good fast convergence, using far less training data, and less computational effort, the three-term backpropagation was adopted.

## Figures and Tables

**Table 1:** The most common fused deposition modelling (FDM) materials.

Material	Density (g/cc)	Young’s Modulus (MPa)	Tensile Strength (MPa)	References
Acrylonitrile-Butadiene-Styrene (ABS)	1.05	2180–2230	26–31	[32,34,35]
Poly Lactic Acid (PLA)	1.27	3368	56	[32,36,37]
Nylon	1.05	1138–1282	28–32	[34,38]
Polycarbonate (PC)	1.24	1958–1944	30–40	[34,38,39]

**Table 2:** Design of Experiment Range Matrix.

Parameters		ABS Core Density	Number of Composite Layers
Level	1	High	1
	0	Medium	2
	−1	Low	3

**Table 3:** As supplied typical fibre properties.

Property	Value	Units
Number of Filaments	3000	
Typical Density	1.79	g/cm^3^
Tow Tensile Strength	4410	MPa
Tow Tensile Modulus	235	GPa
Elongation	1.8	%

**Table 4:** Generated experimental matrix.

Run	Core Density	Number of Layers	Structure Density (g/cc)	Specific Strength (kN·m/kg)	Young’s Modulus (GPa)
-	Low	0	0.47	20	0.63
-	Medium	0	0.56	23	0.76
-	High	0	0.65	19	0.84
1	Medium	3	0.72	116	11.2
2	High	1	0.76	65	4.1
3	Low	1	0.56	68	4.0
4	Medium	1	0.64	75	3.9
5	High	2	0.77	103	7.0
6	Medium	2	0.68	99	6.6
7	Low	3	0.64	145	10.1
8	High	3	0.79	118	10.2
9	Low	2	0.59	73	7.3

**Table 5:** Response surface coefficients values.

Response	$b_{0}$	$b_{1}$	$b_{2}$
$E_{\left(\mathrm{High}\;\mathrm{density}\;\mathrm{infill}\right)}\;$	1.92	1.88	0.3
$E_{\;\left(\mathrm{Medium}\;\mathrm{density}\;\mathrm{infill}\right)}$	0.92	1.85	0.3
$E_{\;\left(\mathrm{Low}\;\mathrm{density}\;\mathrm{infill}\right)}$	2.05	2.44	0.3
${\mathrm{Specific}\;\mathrm{Strength}\;}_{\left(\mathrm{High}\;\mathrm{density}\;\mathrm{infill}\right)}$	62.9	1.8	6.2
${\mathrm{Specific}\;\mathrm{Strength}\;}_{\left(\mathrm{Medium}\;\mathrm{density}\;\mathrm{infill}\right)}$	38.9	13.8	6.2
${\mathrm{Specific}\;\mathrm{Strength}\;}_{\left(\mathrm{Low}\;\mathrm{density}\;\mathrm{infill}\right)}$	76.2	−4.2	6.2
